# Associations between nonrestorative sleep and suicidal ideation: a Japanese general population survey

**DOI:** 10.1038/s41598-025-87897-6

**Published:** 2025-02-07

**Authors:** Kouju Yamada, Yoshiyuki Kaneko, Chisato Konno, Ryuji Furihata, Yuichiro Otsuka, Yoshitaka Kaneita, Makoto Uchiyama, Masahiro Suzuki

**Affiliations:** 1 Department of Psychiatry, Nihon University School of Medicine, 30-1 Oyaguchi-Kamicho, Itabashi-ku, Tokyo, 173-8610 Japan; 2 Kumpukai Yamada Hospital, 3-4-10 Minamicho, Nishitokyo-shi, Tokyo, 188-0012 Japan; 3 Agency for Student Support and Disability Resources, Kyoto University, Yoshida-Honmachi, Sakyo-ku, Kyoto, 606-8501 Japan; 4 Division of Public Health, Department of Social Medicine, Nihon University School of Medicine, 30-1 Oyaguchi-Kamicho, Itabashi-ku, Tokyo, 173-8610 Japan; 5 Tokyo Adachi Hospital, 5-23-20 Hokima, Adachi-ku, Tokyo, 121-0064 Japan

**Keywords:** Sleep, Insomnia, Nonrestorative sleep, Suicidal ideation, Epidemiology, Population surveillance, Risk factors, Comorbidities, Epidemiology

## Abstract

Insomnia symptoms and short sleep duration are known to be associated with suicidal ideation. The aim of this study was to investigate the association between nonrestorative sleep (NRS), a subjective experience of feeling unrefreshed after waking up, and suicidal ideation in the general population. We analyzed data from a cross-sectional survey of 2559 randomly sampled adults living in Japan. The participants were asked about insomnia symptoms (difficulty initiating sleep, difficulty maintaining sleep, and early morning awakening), NRS, sleep duration, and the presence of suicidal ideation. Logistic regression analysis was used to examine the associations among short sleep duration (< 6 h), insomnia symptoms, NRS, and suicidal ideation. Possible confounding factors were adjusted using propensity scores. In the multivariate analysis adjusting for the confounding effects of other sleep-related factors, in addition to psychiatric, sociodemographic, and lifestyle factors, only NRS showed a positive association with suicidal ideation (adjusted odds ratio [aOR] = 2.266). These findings suggest the importance of focusing on NRS for suicide prevention in the general population.

## Introduction

Insomnia symptoms and short sleep duration are known to be associated with suicidal ideation and suicide attempts^1–6^. For example, a cohort study using polysomnography showed that insomnia is associated with an increased risk of suicidal ideation^2^. Interviews with bereaved family members and others have also shown that, compared with control subjects, suicide victims had more insomnia symptoms before they died by suicide^6^. Moreover, a meta-analysis found that sleep disturbances increase the relative risk of suicidal ideation and suicide attempts by two to three times^5^. Nowadays, sleep disturbance is regarded as an important target for suicide prevention, with sleep treatment being reported to reduce suicidal ideation^7^.

In the past, insomnia symptoms and sleep duration have been mainly used to assess the association between poor sleep and various mental and physical problems, including suicide. Meanwhile, nonrestorative sleep (NRS), a subjective experience of feeling unrefreshed after waking up, has been attracting increasing attention in recent years as an indicator for assessing the quality of sleep, based on the idea that a refreshed feeling upon awakening arises as a by-product of the recovery of mental and physical functions^8,9^. Previously, NRS was considered one of the main symptoms of insomnia. However, NRS has come to be regarded as a non-specific symptom found in various sleep disorders, including obstructive sleep apnea, periodic limb movement disorder, shift work sleep disorder, and primary hypersomnia including narcolepsy, and NRS is now considered to indicate a lack of refreshment that encompasses the effects of a variety of sleep disorders^8,9^. There is now a growing belief that NRS has a negative impact on physical and mental health based on findings that it is related to physical and mental disorders such as depressive symptoms^10^, metabolic syndrome^11^, and mortality^12^. However, to our knowledge, no reports have shown a relationship between NRS and suicidal ideation, except for one that investigated this relationship in adolescents^13^. Based on the findings that other sleep disorders besides insomnia that lead to NRS, such as sleep apnea^14^ and narcolepsy^15^, are associated with suicidality, it can be considered that NRS may also be associated with suicidal ideation.

Given this background, in the present study, we analyzed general population data in Japan from before the coronavirus disease 2019 pandemic to examine the relationship between poor sleep, including NRS, and suicidal ideation. We hypothesized that NRS is associated with suicidal ideation independently of other sleep-related factors such as insomnia symptoms and sleep duration.

## Methods

### Participants

This study was a part of the Nihon University Sleep and Mental Health Epidemiology Project (NUSMEP)^16–21^, a survey aimed at determining the factors associated with depressive symptoms in the general Japanese population. NUSMEP was conducted as a part of an omnibus survey in August and September 2009 through a commission to a polling agency (Central Research Services, Tokyo, Japan). Details of the data collection methods were described in our previous reports^17,20^. In total, 2559 individuals participated in the present survey. Informed consent was obtained from all the study participants The distribution patterns in our sample and Japan Census data from 2008 seemed comparable with respect to sex and age distribution^20^. This study was approved by the ethics committee of the Nihon University School of Medicine (2023-08). All experiments were performed in accordance with relevant guidelines and regulations.

### Procedures

The questionnaire used in the present survey consisted of 77 items, including (1) sociodemographic and lifestyle information, (2) information about psychiatric features, and (3) sleep measures.

#### Sociodemographic and lifestyle variables

The sociodemographic variables analyzed in the present study included sex, age (20–39, 40–59, or ≥ 60 years), educational achievement (junior high school, senior high school, and college or higher), marital status (married or unmarried), subjective economic status (upper, middle, or lower), and community size (a big city with a population of ≥ 150,000, a small city with a population of < 150,000, or a town or village), based on our previous studies^16,17,20^. In addition, employment status (employed, student or homemaker, or unemployed) was included in the analysis as a sociodemographic variable based on previous studies that reported a relationship between unemployment and suicidal ideation^22,23^.

The lifestyle variables included current smoker and alcohol drinker. The following question was used to identify current smokers: “Have you ever smoked?” (Never/Not in the last month/Fewer than 100 cigarettes in the past 6 months/More than 100 cigarettes in the past 6 months). For this question, “Fewer than 100 cigarettes in the past 6 months” or “More than 100 cigarettes in the past 6 months” was considered to indicate a current smoker. The following question was used to identify alcohol drinkers: “Do you drink alcoholic beverages more than three times per week, comprising more than one glass of sake (180 mL)?” (Yes/Not so much/Not at all). A glass of sake is equal to a 500-mL bottle of beer, 80 mL of distilled spirits, 60 mL of whiskey, or two glasses of wine (240 mL). For this question, “Yes” was considered to indicate an alcohol drinker.

#### Information about psychiatric features

We used the CES-D^24^ to evaluate the participants’ depressive symptoms. We used the Japanese version of the CES-D^25^, which is a 20-item inventory designed specifically to assess depressive symptoms in the general population, to screen for current depressive states during the 1-week period preceding the survey. Each item on the CES-D is scored from 0 to 3, yielding a total score ranging from 0 to 60, with higher scores indicating more severe depressive symptoms. The mean CES-D scores sorted by sex and age were described in our previous report^20^. Participants with CES-D scores ≥ 16 were defined as having depressive tendencies based on previous studies showing that a CES-D score ≥ 16 is highly suggestive of clinical depression^24,25^.

The following question was used to identify the presence of suicidal ideation: “In the past 2 weeks, have you thought about harming yourself or dying by suicide, or have you repeatedly thought that you would be better off dead?” (Yes/No).

The participants’ personality features were assessed using the Japanese version of the short-form of the Eysenck Personality Questionnaire-Revised (EPQ-R). The EPQ-R consists of a questionnaire that assesses personality traits dimensionally^26^. In the present study, the sum score of 12 items on the EPQ-R related to neuroticism (EPQ-RN), which has been shown to be associated with depression^27^, was calculated. Depending on the median EPQ-RN score across participants, the same as in our previous study^19^, participants with an EPQ-RN score ≥ 2 were defined as having neurotic tendencies.

The following question was used to identify the presence of perceived stress: “Have you felt stressed in your daily life during the past month?” (Very/Somewhat/Not very/Not at all). Participants responding “Very” or “Somewhat” were defined as perceiving high stress levels.

#### Sleep measures

We drew up five questions to examine sleep disturbance patterns in the participants, with reference to the Japanese version of the Pittsburgh Sleep Quality Index^28^. The following questions about insomnia symptoms during the previous month were included in the questionnaire:

- “How often have you had difficulty falling asleep (difficulty initiating sleep [DIS])?”
- “How often have you woken up frequently at night (difficulty maintaining sleep [DMS])?”
- “How often have you woken up too early in the morning (early morning awakening [EMA])?”

Each question had four possible responses: “Not at all,” “Less than once a week,” “Once or twice a week,” or “Three or more times a week.” For these questions, “Once or twice a week” and “Three or more times a week” were considered affirmative answers. Affirmative answers to the questions were considered to indicate the presence of DIS, DMS, or EMA.

The following question was used to identify the presence of NRS: “Do you feel rested after a typical night’s sleep?” (Very/Somewhat/Not very/Not at all). Participants who answered “Not very” or “Not at all” were defined as having NRS.

The following question about sleep duration during the previous month was also included in the questionnaire: “How many hours and minutes of actual sleep do you get at night (sleep duration)?”. A sleep duration < 6 h was defined as a short sleep duration.

### Statistical analysis

Comparisons of sleep-related factors between participants with suicidal ideation and those without suicidal ideation were performed using chi-square tests. Logistic regression analyses were used to assess the associations between suicidal ideation and sleep disturbances. Initially, we examined all variables in univariate models. To assess the associations, sets of logistic regression analyses with each sleep-related factor as an exploratory variable were performed. A series of univariate regression analyses was conducted with suicidal ideation as an objective variable. After the univariable regression analyses, we performed multivariate logistic regression analyses to adjust for the confounding effects of sociodemographic factors (sex, age, educational achievement, marital status, subjective economic status, community size, and employment status), lifestyle (current smoker and alcohol drinker), and psychiatric factors (depressive tendencies, neurotic tendencies, and perceived stress) (Adjusted 1). We then performed an additional multiple logistic regression analysis for all items that showed a significant association in the multivariate models (Adjusted 1) to assess the confounding effects of other sleep-related factors in addition to sociodemographic, lifestyle, and psychiatric factors (Adjusted 2). To adjust for the effects of possible confounding factors in these multiple logistic regression analyses, we used propensity scores, since the number of possible confounding factors is large in relation to the number of participants with suicidal ideation^29^. An individual propensity score was calculated for each patient by logistic regression analysis with each sleep-related factor as a response variable and with possible confounding factors as exploratory variables. The calculated propensity scores were included in the logistic models (Adjusted 1 and 2) as exploratory variables. Statistical significance was set at 0.05. All analyses were performed using SPSS version 28 (IBM, Armonk, NY, USA).

## Results

### Demographic data and associations with suicidal ideation

The male/female ratio was comparable between the groups with and without suicidal ideation (Table 1). The proportion of participants aged 40–59 years was higher among those with than without suicidal ideation, whereas the proportion of participants aged 20–39 years was lower among those with than without suicidal ideation. No participants with suicidal ideation had an upper subjective economic status.

**Table 1:** Associations between demographic characteristics and suicidal ideation.

Variable	Suicidal ideation (n = 65)		No suicidal ideation (n = 2494)	
	n	%	n	%
Sex				
Male	33	50.8	1130	45.3
Female	32	49.2	1364	54.7
Age (y)				
20–39	12	18.5	757	30.4
40–59	29	44.6	832	33.4
≥ 60	24	36.9	905	36.3
Educational achievement				
Junior high school	16	24.6	338	13.6
High school	33	50.8	1305	52.3
College or higher	16	24.6	851	34.1
Marital status				
Married	50	76.9	1837	73.7
Unmarried	15	23.1	657	26.3
Subjective economic status				
Upper	0	0.0	52	2.1
Middle	48	73.8	2077	83.3
Lower	17	26.2	365	14.6
Community size				
Big city	16	24.6	654	26.2
Small city	41	63.1	1591	63.8
Town or village	8	12.3	249	10.0
Employment status				
Employed, student, or homemaker	50	76.9	2094	84.0
Unemployed	15	23.1	400	16.0
Current smoker	28	43.1	1310	52.5
Alcohol drinker	12	18.5	587	23.5
Depressive tendencies	15	23.1	163	6.5
Neurotic tendencies	26	40.0	462	18.5
Perceived stress	41	63.1	1238	49.6

### Prevalence of NRS and other sleep-related symptoms

NRS was present in 19.1% of all participants with no significant suicidal ideation, which was comparable to the prevalence of other symptoms (Table 2). Among the participants with significant suicidal ideation, the prevalence of NRS was 43.1%. The prevalence of all sleep-related symptoms was higher in participants with suicidal ideation than in those with no significant suicidal ideation.

**Table 2:** Differences of sleep-related factors between participants with and without suicidal ideation.

	Suicidal ideation		No suicidal ideation		χ^2^	p
	n	%	n	%		
DIS	22	33.8	357	14.3	19.2	< 0.001
DMS	25	38.5	655	26.3	4.8	0.033
EMA	13	20.0	286	11.5	4.5	0.035
Short sleep duration	18	27.7	447	17.9	4.1	0.044
NRS	28	43.1	476	19.1	23.1	< 0.001

*DIS* difficulty initiating sleep, *DMS* difficulty maintaining sleep, *EMA* early morning awakening, *NRS* nonrestorative sleep.

### Association between sleep-related symptoms and suicidal ideation

In the univariate logistic regression analyses of all participants, all sleep-related factors were shown to be associated with suicidal ideation (Table 3). After adjusting for the confounding effects of psychiatric, sociodemographic, and lifestyle factors (Adjusted 1), only DIS (adjusted odds ratio [aOR] = 2.214, p = 0.008) and NRS (aOR = 2.474, p = 0.001) showed significant associations with suicidal ideation. In the multivariate logistic regression models that included other sleep-related factors in addition to sociodemographic, lifestyle, and psychiatric factors (Adjusted 2), only NRS (aOR = 2.266, p = 0.008) showed a significant association with suicidal ideation.

**Table 3:** Associations between suicidal ideation and sleep-related factors in all participants.

	Crude			Adjusted 1^a^			Adjusted 2^b^		
	aOR	95% CI	p	aOR	95% CI	p	aOR	95% CI	p
DIS	3.063	1.810–5.182	< 0.001	2.214	1.235–3.971	0.008	1.769	0.892–3.511	0.103
DMS	1.755	1.056–2.915	0.030	1.297	0.752–2.235	0.350			
EMA	1.930	1.038–3.588	0.038	1.334	0.685–2.596	0.397			
Short sleep duration	0.570	0.328–0.991	0.046	1.319	0.730–2.381	0.359			
NRS	3.208	1.944–5.295	< 0.001	2.474	1.428–4.285	0.001	2.266	1.236–4.154	0.008

*aOR* adjusted odds ratio, *CI* confidence interval, *DIS* difficulty initiating sleep, *DMS* difficulty maintaining sleep, *EMA* early morning awakening, *NRS* nonrestorative sleep. ^a^Adjusted for sociodemographic, lifestyle, and psychiatric factors. ^b^Adjusted for other sleep-related factors in addition to sociodemographic, lifestyle, and psychiatric factors.

## Discussion

The results of the present study indicated a relationship between NRS and suicidal ideation, with the participants with NRS more likely to report suicidal ideation. To the best of our knowledge, this is the first study to investigate the association between NRS and suicidal ideation in the general adult population.

Suicidal ideation has been shown to be related to trait neuroticism^30^, stress^31^, depression^32,33^, alcohol and tobacco use^34,35^, and sociodemographic factors such as unemployment^23^ or low economic status^36^. Therefore, we included these factors as covariates in regression models and found a significant relationship between NRS and suicidal ideation. Our results cannot be explained by a confounding effect of sociodemographic or psychiatric factors, which suggests that NRS is independently related to suicidal ideation. These results are in line with a previous cross-sectional study involving Korean adolescents that found a relationship between NRS and suicidal ideation^13^.

Our finding that NRS is related to suicidal ideation implies that, given the hypothesis that a refreshed feeling upon awakening is a by-product of brain recovery during sleep^8,9^, people may be more likely to experience suicidal ideation when they have inadequate recovery. This implication is supported by a finding from a longitudinal case–control cohort study that NRS is associated with an elevated risk of death by suicide at a 10-year follow-up of community-dwelling older adults^37^. In the present study, no significant relationship was found between insomnia symptoms or sleep duration and suicidal ideation in a model in which insomnia symptoms and sleep duration were included with NRS. Given that adequately long and well-maintained sleep leads to brain recovery from fatigue, whether fatigue is well recovered by sleep may be associated with the presence or absence of suicidal ideation.

Regarding an explanation for the link between sleep problems such as insomnia and suicidal behavior, a model has been proposed in which sleep problems lead to sleep loss, which in turn, causes damage to frontal lobe function and difficulties in social life such as being late for work and making mistakes at work because of sleepiness, possibly leading to suicidal ideation^38^. Therefore, NRS may be a useful indicator of sleep disturbance leading to biological or sociopsychological changes that can possibly cause suicidal ideation. Further research is needed to investigate the relationships between NRS and frontal lobe function and social life, since this may lead to a better understanding of the mechanisms by which disturbances in the recovery process can lead to suicidal ideation.

However, contrary to the considerations noted so far, it is also possible that NRS occurs because of a mental state that causes suicidal ideation. Negative emotions such as anxiety and anger, which often co-occur with suicidal ideation^39,40^, may suppress sleep and prevent refreshment^41^. Further longitudinal studies are needed to clarify the details of the causal relationship between suicidal ideation and NRS.

The present study had several methodological limitations. First, as previously mentioned, because this study was cross-sectional in design, causal relationships could not be determined. It is not possible to determine from this study whether NRS causes suicidal ideation or whether NRS can predict suicidal ideation. Second, psychiatric attendance and the use of psychiatric medications, which may affect insomnia symptoms, sleep duration, and NRS, were not assessed in this study. Third, in this study, we used original criteria to identify NRS. At the time the data were collected, there were few commonly used criteria for NRS in sleep research. Therefore, a proprietary question was used to detect participants with characteristics of NRS. In recent years, questionnaires to identify NRS have started to be developed^42^, and it is hoped that the validity of the present results will be re-evaluated using such a questionnaire in the future. Fourth, data from 2009 were used in this study for the purpose of assessing the population unaffected by the coronavirus disease 2019 pandemic. It would be desirable to revisit the results of this study using recent general population data, since current individual characteristics may differ from those prior to the COVID-19 pandemic. Fifth, sleep-related factors were assessed by self-report in this study. Because self-reports of sleep symptoms often differ from objective measures^43^, it is desirable to examine the relationships between objective measures and suicidal ideation and compare them to the relationships with subjective measures. Finally, since the following question was used in this study to identify the presence of suicidal ideation: “In the past 2 weeks, have you thought about harming yourself or dying by suicide, or have you repeatedly thought that you would be better off dead?”, those with a desire to self-harm, but without suicidal ideation, may be categorized as having suicidal ideation. Future studies may be needed to distinguish between desire to self-harm and suicidal ideation.

## Conclusion

The results of the present study indicated that individuals with NRS tend to report suicidal ideation. This tendency was not explained by insomnia symptoms or a short sleep duration. These findings suggest the importance of focusing on NRS for suicide prevention in the general population.

## Data Availability

The raw data supporting the conclusions of this manuscript will be made available by one of the corresponding authors without undue reservation, to any qualified researcher.

## Abbreviations

aOR: Adjusted odds ratio
CES-D: Center for epidemiologic studies depression scale
DIS: Difficulty initiating sleep
DMS: Difficulty maintaining sleep
EMA: Early morning awakening
EPQ-RN: Eysenck personality questionnaire-revised neuroticism
NRS: Nonrestorative sleep
NUSMEP: Nihon University Sleep and Mental Health Epidemiology Project
